# Governing for sustainable health: institutional pathways in China's sport and health policy (1949–present)

**DOI:** 10.3389/fpubh.2025.1660488

**Published:** 2025-08-29

**Authors:** Guanqun Luo

**Affiliations:** College of Education, Zhejiang University, Hangzhou, China

**Keywords:** historical institutionalism, sport and health policy, health management transition, collaborative governance, precision weight management, Healthy China 2030

## Abstract

**Introduction:**

Since 1949, China's sport and health policies have evolved from mass-mobilization campaigns centered on “strengthening physical fitness” to a data-driven framework emphasizing “weight management” and cross-sectoral collaboration under the Healthy China 2030 strategy. However, the institutional processes underpinning this transition remain insufficiently understood and rarely subjected to systematic analysis.

**Methods:**

Guided by historical institutionalism, this study conducts a discourse analysis of 15 national policy documents issued between 1949 to present. Python-based text segmentation and keyword frequency and co-occurrence analyses were used to trace shifts in governance instruments and institutional layering.

**Results:**

Findings reveal a three-stage trajectory: (1) an initial stage of ideological mobilization (1949–1995) characterized by centralized fitness directives; (2) a second stage (1996–2015) featuring chronic disease prevention, performance-based targets, and provincial pilot programs; and (3) a current stage (2016-present) focused on obesity control, digital health platforms, and real-time multi-level coordination.

**Discussion:**

This path—dependent layering-from campaign rhetoric to target-based planning to data-enabled governance—demonstrates how embedding new health objectives within entrenched institutional routines, while incrementally integrating cross-sectoral data systems, can foster sustainable policy adaptation. These insights offer a practical and transferable model for other middle-income countries pursuing scalable, evidence-informed health governance.

## 1 Introduction

Over the past few decades, global public health governance has undergone profound transformations. The rising burden of non-communicable diseases (NCDs), accelerated population aging, and increasingly sedentary lifestyles have repositioned sport as a core instrument for health promotion and disease prevention (1–3). In developed countries, fitness legislation, community-based programs, and cross-sectoral governance have become institutionalized, embedding physical activity policies within broader public health agendas (4). The Organization for Economic Co-operation and Development (OECD) has further underscored the role of digital technologies and intersectoral collaboration in addressing chronic disease and health inequality (5, 6). Yet in many development countries, institutional inertia, governance fragmentation and resource constraints persist, raising the question of how states adapt to emerging health challenges within existing institutional arrangements (7).

China provides an instructive case. Since 1949, sport policy has been closely aligned with national development goals. The early slogan “Develop sports and enhance the people's physique” reflected a mass-mobilization strategy aimed at strengthening physical fitness and national reconstruction. The enactment of the Sports Law and the launch of the National Fitness Program Outline in 1995 marked a shift toward legalized, organizational governance. The Healthy China 2030 Planning Outline, released in 2016, further repositioned sport as a strategic public health tool, signaling a transition from fitness enhancement to health-centered, integrated governance (8). Despite these advances, new challenges have emerged. By 2024, China's life expectancy had reached 79 years (9, 10) yet obesity had become a major public health concern: 16.4% of adults were obese, while 11.1% and 7.9% of children and adolescents aged 6–17 were overweight and obese, respectively (11). In response, 2024 was designated the “Year of Weight Management,” emphasizing cross-sector collaboration, digital technology, and personalized health management, embedding sport policy more firmly within chronic disease prevention and public health governance.

Existing scholarship provides valuable insights into these dynamics. International research highlights the importance of cross-sectoral governance (12), institutional layering and incremental adjustment (13), and the political logic of policy and institutional reform (14). Studies on developing countries stress resource limitations, administrative fragmentation, and weak policy integration (7). In China, research has focused on three main areas: macro-level interpretations of the Healthy China strategy (15, 16), analyses of the institutional logic and policy pathways of “sports–medicine integration” (17, 18), and policy responses to youth fitness decline and chronic disease prevention (19). While these studies shed light on the strategic value and practical challenges of sport and health policy, they reveal two key gaps: a lack of long-term, systematic analysis from an institutional evolution perspective, and limited exploration of mechanisms-such as institutional tensions, tool embedding, and actor adaptation-that explain how gradual reform occurs within a highly centralized governance system.

Against this backdrop, this study addresses two questions:

*How have China's sport and health policies evolved institutionally since 1949? And through what mechanisms have they adapted to governance challenges?*

To answer these questions, we employ a historical institutionalist framework emphasizing path dependence, critical junctures, and gradual change (20). We propose a three-level analytical model: the macro level traces shifts in national strategic goals; the meso level examines the evolution of institutional design and governance tools; and the micro level analyzes actor strategies and practices. Methodologically, we analyze 15 national-level policy documents issued between 1949 and 2024, applying Python-based textual analysis to extract semantic patterns, identify institutional logics across different periods, and classify policy evolution into distinct stages.

This study makes three contributions. First, it introduces the “institutional tensions–nested evolution-actor adaptation” model to explain China's sport health governance adaptation mechanisms. Second, it integrates textual semantic analysis with historical institutionalism, bridging “text” and “institutional logic” in the empirical study of policy change. Third, it draws lessons from China's experience for other large developing countries seeking to reconstruct public health governance and pursue coordinated reforms amid rapid social transformation.

## 2 Theoretical framework and analytical approach

### 2.1 Institutionalism and the Logic of ”change within stability“

In a highly bureaucratic governance system dominated by a strong state, policy change is often not characterized by abrupt ruptures but by incremental adjustments embedded within existing institutional tracks. This phenomenon of “change within stability” (21) can be effectively understood through the lens of institutionalism. Institutional theory offers three main variants: rational choice institutionalism, sociological institutionalism, and historical institutionalism. While the first two have strong explanatory power in specific contexts, they are less suited for analyzing the long-term evolution of China's sport and health policies. Rational choice institutionalism emphasizes individual rational decisions shaped by incentive structures, making it better for explaining short-term decisions and institutional design. Sociological institutionalism highlights cultural norms and legitimacy but often underplays the internal power struggles and tension mechanisms within institutions. By contrast, historical institutionalism focuses on the persistence and constraining effects of institutions over time (22), making it particularly effective in explaining the coexistence of gradual evolution and path dependence—thus serving as the most appropriate perspective for understanding the institutional trajectory of China's sport and health governance.

### 2.2 Core propositions of historical institutionalism and their relevance

Historical institutionalism emphasizes both the enduring nature and gradual transformation of institutions. Three core propositions are particularly relevant:

- Path dependence: Early institutional choices are reinforced through feedback mechanisms, shaping the boundaries of subsequent policy change. Since the adoption of the national strategy of “enhancing the people's physique” in 1949, China‘s sport policy has long retained a mobilization logic centered on physical fitness.
- Critical junctures: Major political or social turning points create “windows of opportunity” for institutional adjustment. The enactment of the Sports Law and the launch of the National Fitness Program Outline in 1995 ushered sport policy into a legalized and institutionalized phase; the release of the Healthy China 2030 Planning Outline in 2016 further promoted the deep integration of sport and health governance.
- Gradual evolution: Institutional change often takes the form of nested amendments and the layering of policy tools, rather than wholesale replacement. For example, the recent emergence of health monitoring systems and digital governance platforms has not dismantled existing administrative structures but has extended and innovated within them.

These propositions illuminate the dual forces of institutional inertia and evolutionary logic, providing analytical tools for understanding the phased transformation of China's sport and health governance.

### 2.3 A three-dimensional analytical framework: macro–meso–micro institutional evolution

**Figure 1:**
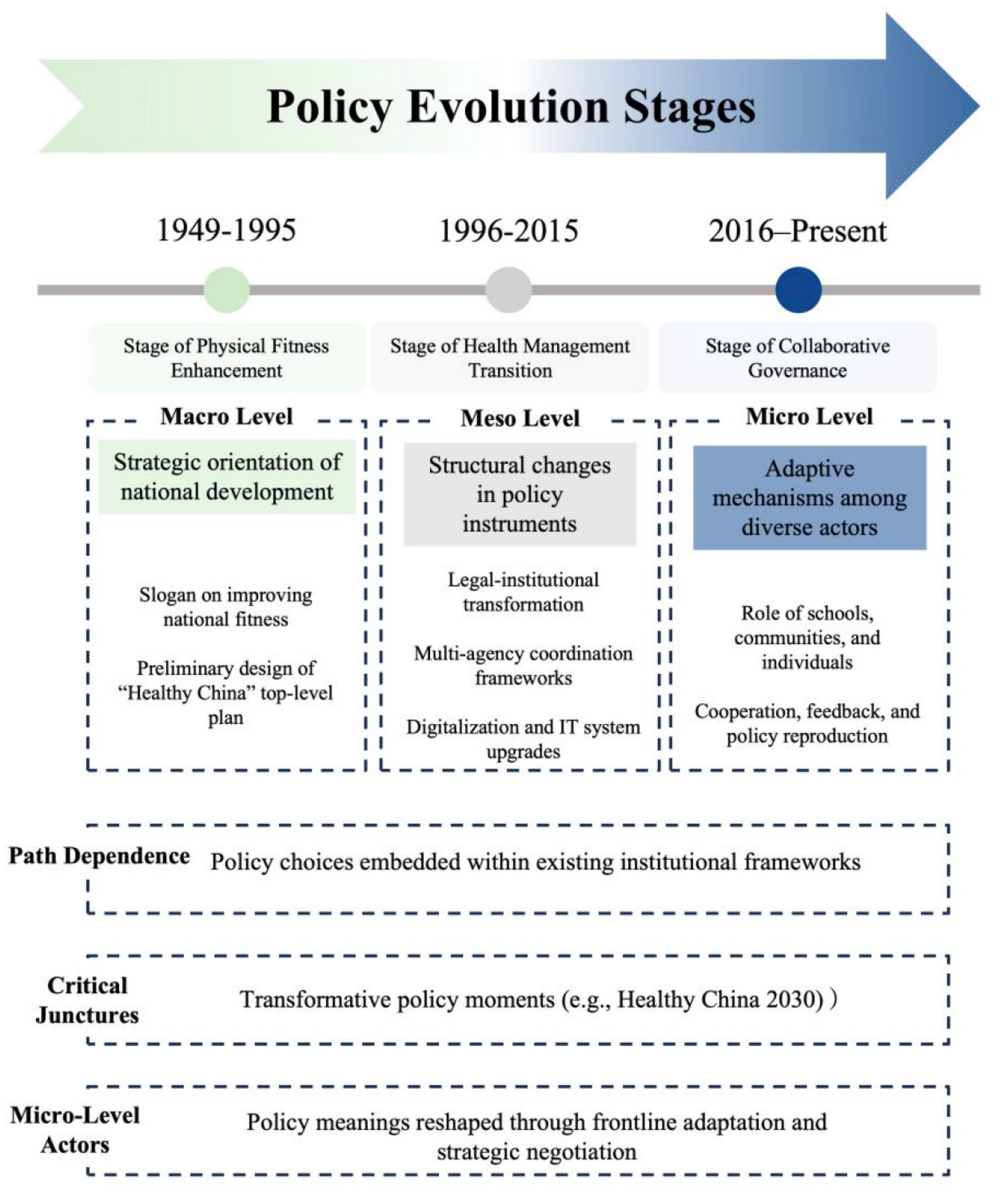
Policy evolution stages and institutional mechanisms in sport and health governance (1949–present).

Building on historical institutionalism, this study proposes a three-dimensional analytical framework—macro, meso, and micro levels—to capture the multi-layered logic of institutional evolution (see Figure 1):

- Macro level: Focuses on shifts in national strategy and ideology, revealing the strategic progression from “enhancing physical fitness” to “Healthy China.”
- Meso level: Examines incremental adjustments in governance tools and institutional design, such as the establishment of laws and regulations, the construction of cross-sector coordination mechanisms, and the introduction of performance evaluation systems and digital platforms (23).
- Micro level: Highlights adaptive practices in policy implementation by actors including local governments, schools, communities, and enterprises, showing how institutional reproduction occurs under the combined influences of incentives, accountability, and cultural orientation.

This framework underscores that institutional evolution is not a mere structural transformation but the result of interactive bargaining among multi-level actors operating within specific institutional opportunity structures.

## 3 Methodology and corpus construction

### 3.1 Data sources and policy text selection

To uncover the institutional evolution of China's sport and health policies, this study selects 15 policy documents issued at the national level between 1949 and 2024 as the primary corpus. These documents cover strategic plans, laws and regulations, action programs, and technical standards. They include both programmatic policies-such as the Sports Law and the Healthy China 2030 Planning Outline-and targeted implementation plans, such as the National Student Physical Health Standards and the Implementation Plan for the Prevention and Control of Obesity in Children and Adolescents. The selection was guided by three criteria:

- Authoritativeness: Documents are issued by core central authorities such as the State Council, the National People's Congress, the General Administration of Sport, and the National Health Commission, ensuring institutional legitimacy.
- Representativeness: The corpus includes both strategic and operational documents, capturing the core policy objectives and governance orientations of each period.
- Sustainability: Selected documents demonstrate continuity and enforceability rather than being short-term or one-off policy declarations. For example, the National Fitness Program has been periodically revised across different years, reflecting its long-term character and institutional embeddedness.

Applying these criteria ensures that the corpus systematically reflects the institutional logic, governance tools, and strategic evolution of China's sport and health policies across historical periods, focusing on national-level policy documents for their authority and strategic relevance.

### 3.2 Text processing and semantic analysis

The textual analysis was conducted in Python 3.11. First, the jieba segmentation tool was applied to tokenize the corpus, using four Chinese stopword lists from Harbin Institute of Technology, Baidu, the Chinese Academy of Sciences, and Tsinghua University, supplemented with a custom exclusion list to remove function words, punctuation, and numeric-only tokens. Second, based on the theoretical framework and existing studies, a whitelist of 46 keywords was constructed, covering semantic categories such as governance tools (e.g., “platform,” “monitoring,” “system”), institutional logic (e.g., “coordination,” “integration,” “mechanism”), and strategic objectives (e.g., “health,” “youth,” “management”). Finally, matplotlib was used to generate heatmaps and trend charts of keyword frequencies, visually illustrating the historical trajectory of policy semantics.

### 3.3 Institutional stage division and logic identification

**Figure 2:**
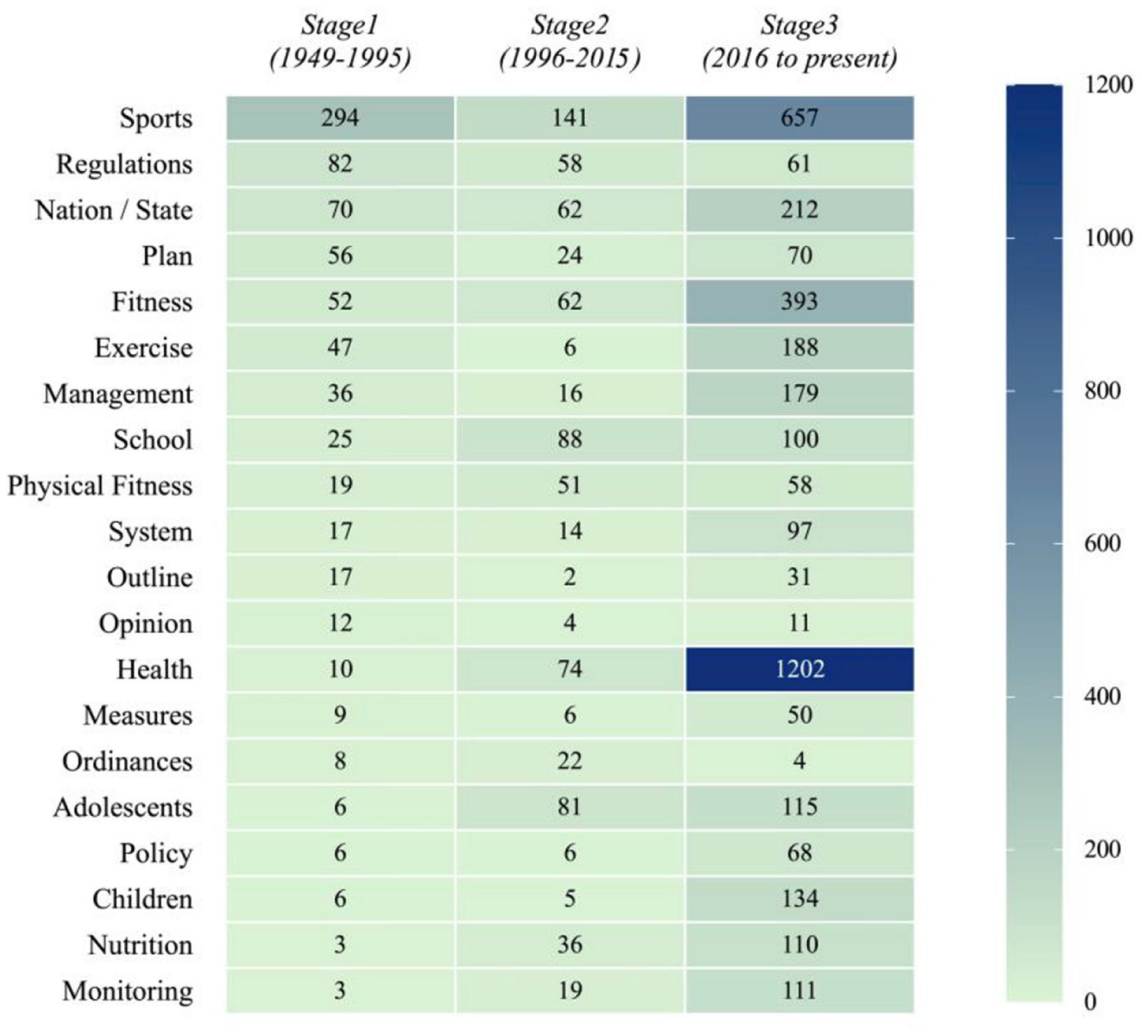
Keyword heatmap: a heatmap displaying the frequency distribution of 46 core terms across three policy phases (1949–1995; 1996–2015; 2016–present). Colors range from **light** to **dark** to indicate low to high term frequency, with darker regions highlighting periods in which a given keyword was particularly prevalent, thereby visualizing the semantic focus shifts over time.

Drawing on semantic trends, historical context, and close reading of policy texts, this study divides the evolution of China's sport and health policies into three stages (see Table 1 and Figure 2).

**Table 1:** Evolution of core policy documents in China's Sports and Health Governance (1949–present).

**Phase**	**Time period**	**Core objectives**	**Policy documents/sources**
Stage 1: Physical Fitness Enhancement	1949–1995	Establish a state-led sports system and improve national physical fitness	Central Government of China. (1952). Develop sports and enhance people's physique [In Chinese].
			National Sports Commission. (1982). National Physical Exercise Standards [In Chinese].
			General Administration of Sport of China. (1995a). Olympic Glory Plan [In Chinese].
			General Administration of Sport of China. (1995b). National Fitness Program Outline [In Chinese].
			National People's Congress. (1995). Sports Law of the People's Republic of China [In Chinese].
Stage 2: Health Management Transition	1996–2015	Transition from sports to health management with a focus on chronic disease prevention	State Council of China. (2007). Opinions on strengthening youth sports and improving adolescent physical health [In Chinese].
			State Council of China. (2009). National Fitness Regulations [In Chinese].
			Ministry of Education. (2014). National Student Physical Health Standards (Revised 2014) [In Chinese].
			National Health and Family Planning Commission. (2015). Report on the nutrition and chronic diseases of Chinese residents (2015) [In Chinese].
Stage3: Collaborative Governance Phase	2016–present	Promote the Healthy China strategy through interdepartmental collaboration, with a focus on obesity and precision management	State Council of China. (2016a). Healthy China 2030 Planning Outline [In Chinese].
			General Administration of Sport of China. (2016). National Fitness Plan (2016–2020) [In Chinese].
			State Council of China. (2019a). Healthy China Action (2019–2030) [In Chinese].
			General Administration of Sport of China. (2019b). Outline for Building a Leading Sports Nation [In Chinese].
			National Health Commission. (2020). Implementation plan for the prevention and control of obesity in children and adolescents [In Chinese].
			General Administration of Sport of China. (2021). National Fitness Plan (2021–2025) [In Chinese].
			National People's Congress. (2022). Sports Law of the People's Republic of China (2022 Revision) [In Chinese].
			National Health Commission. (2024). Guidelines on dietary intervention for adult obesity (2024 Edition) [In Chinese].

These three phases not only demarcate changes in policy focus and actor constellation, but also represent institutional shifts in the underlying governance model-from mobilization-based systems to platform-based, precision governance.

Stage 1 (1949–1995): Physical Mobilization and Institutional Formation.

Heatmap analysis shows that high-frequency keywords in this stage include sports, regulations, and nation/state. “Sports” ranks as the most frequent term in the entire corpus, reflecting the state's persistent emphasis on sport as a core tool for physical mobilization and nation-building. “Regulations” shows a marked rise in the 1980s, corresponding to the promulgation of the National Physical Exercise Standards in 1982, signaling the gradual institutionalization of legal norms. Representative documents include the National Physical Exercise Standards (1982) and the Sports Law (1995). The institutional logic of this period centered on establishing sport's strategic status in national governance through administrative mobilization and legal codification.

Stage 2 (1996–2015): Transition to Health Management.

Heatmap results indicate notable increases in the keywords school, adolescents, and physical fitness. The frequency of “school” and “adolescents” nearly doubled compared to the previous stage, reflecting the growing integration of the education system and the prominence of youth health issues. “Physical fitness” peaks sharply around 2007 and 2014, aligning with the release of the Opinions on Strengthening Youth Sports (2007) and the revised National Student Physical Health Standards (2014), signaling a shift from fitness enhancement to chronic disease prevention and youth health promotion. Other representative documents include the Report on Nutrition and Chronic Disease of Chinese Residents (2015). Governance logic during this period featured the legalization of policy tools, multi-department coordination, and an emerging orientation toward health management.

Stage 3 (2016–present): Collaborative Governance and Precision Intervention.

The heatmap reveals a sharp surge in “health” after 2016, making it the most salient keyword in the corpus. Terms such as “management,” “nutrition,” and “monitoring” also emerge and trend upward, indicating a governance shift toward cross-sector collaboration and targeted interventions. Representative documents include the Healthy China 2030 Planning Outline (2016), the Healthy China Initiative (2019–2030), and the Implementation Plan for the Prevention and Control of Obesity in Children and Adolescents (2020). The institutional logic of this stage features diversification of governance actors, refinement of governance tools, and the adoption of life-course-oriented governance objectives, embedding sport firmly within the institutional framework of the Healthy China strategy.

### 3.4 Summary

The textual semantic analysis and stage division reveal that the institutional evolution of China's sport and health policies has not followed a linear trajectory but has instead exhibited marked characteristics of path dependence and incremental adjustment. Between 1949 and 1995, the high frequency of the keywords sports and regulations underscored sport's role as a national tool for physical mobilization. From 1996 to 2015, the rise in education- and youth-related terms reflected a policy shift toward health management and chronic disease prevention. Since 2016, the frequent appearance of health and related governance terms indicates that sport has become fully embedded within the Healthy China strategy, achieving cross-sectoral collaboration and precision governance. This stage division not only illustrates the semantic shifts in policy texts but also reflects the logic of path dependence, critical junctures, and gradual evolution emphasized by historical institutionalism, thereby laying the groundwork for subsequent discussions on institutional tensions and the evolution of governance mechanisms.

## 4 Institutional stages and the logic of evolution

### 4.1 Physical mobilization and institutional formation (1949–1995)

#### 4.1.1 Macro level: national strategy and health foundations

In the early years of the People's Republic of China, sport was embedded in nation-building as both a means of improving public fitness and a tool for political mobilization (24). Guided by the socialist planned economy and an emphasis on collective discipline, it functioned as a mass mobilization mechanism and a symbol of socialist modernization. By the early 1990s, amid the Reform and Opening-up, the state's legitimacy increasingly rested on socio-economic development, with the 1993 Olympic bid reflecting a more internationally engaged posture. Against this backdrop, the Sports Law and National Fitness Program Outline of 1995 marked not only administrative reform but also a political decision to anchor sport in the rule-of-law framework and align it with the broader goal of building a socialist market economy, signaling a shift from campaign-style mobilization to legislatively grounded governance.

#### 4.1.2 Meso level: administrative mobilization and institutional prototypes

During this period, sport governance operated within a highly centralized administrative system, forming a vertical mobilization model of “state-led, mass participation” (25). The 1958 Preparedness for Labor and Defense Sports System (commonly known as the Laowei System) standardized physical fitness testing, daily exercise, and defense-related sports, representing one of the earliest measures toward institutionalizing sport. Following China's restoration of its International Olympic Committee membership in 1979, the principles of “combining mass participation with elite performance” and “society-led sport development” were adopted, fostering a conceptual shift in mass sport. In 1982, the Constitution enshrined “enhancing the people's physique” as a state mandate, accompanied by the promulgation of the National Physical Exercise Standards, which established a unified nationwide fitness testing system. In the early 1990s, the Olympic Glory Program elevated elite sport to a central component of national development strategy (26), creating a dual-track system in which mass sport and elite sport developed in parallel.

#### 4.1.3 Micro level: everyday practice and cultural diversification

At the grassroots level, sport activities were widely practiced in schools, workplaces, and rural communities. Radio calisthenics, workplace exercises, employee sports meets, tug-of-war competitions, and basketball tournaments served both to improve physical fitness and to strengthen organizational identity (27). In the early reform era, workplace sport organizations were restored, and rural areas widely established “sport and cultural centers” and “sport and cultural stations” to provide stable venues for community-based sport (28). Local governments also explored diversified development paths. In Shanghai, for example, the 1978 Municipal Sports Work Conference designated school sport as a strategic priority; in 1983, the city launched the Xinmin Evening News Cup youth football tournament and hosted the 5th National Games, thereby advancing both facility construction and public participation (29).

Between 1949 and 1995, China's sport policy completed its institutional foundation through the multi-level interaction of macro strategies, meso-level institutional design, and micro-level practices. Improvements in public health, a vertically mobilized administrative system, and culturally diverse sport forms together facilitated the social embedding of sport. The enactment of the Sports Law in 1995 marked the transition of sport development into a legalized and institutionalized stage, laying the groundwork for the subsequent shift toward health management.

### 4.2 Transition to health management (1996–2015)

#### 4.2.1 Macro level: expanding strategic goals and establishing a national health agenda

By the mid-1990s, China entered a stage characterized by rapid population aging and a significantly heavier burden of non-communicable diseases (NCDs) (30). Against this backdrop, sport policy shifted from a singular logic of “enhancing physical fitness” toward a comprehensive agenda centered on health promotion and chronic disease prevention. The Sports Law and the National Fitness Program Outline, both issued in 1995, established the legal status and strategic significance of nationwide fitness. The 2007 Opinions of the CPC Central Committee and the State Council on Strengthening Youth Sports elevated youth physical health to the level of national strategy, while the 2014 revision of the National Student Physical Health Standards reinforced school responsibilities. The 2015 Report on Nutrition and Chronic Disease of Chinese Residents formally integrated chronic disease prevention into the sport health policy framework. At the macro level, policy objectives expanded from improving physical fitness to encompassing public health management for the entire population.

#### 4.2.2 Meso level: institutionalization and multi-sector system building

The 2009 National Fitness Regulations placed fitness promotion on a normalized, legally grounded track, while education, sport, and health departments strengthened collaboration in areas such as fitness monitoring and health interventions. By 2015, China had over 1.7 million sports venues, with an average of 1.57m^2^ per person; more than 50% of cities and counties had established national fitness centers, and the Farmers' Fitness Project covered 74% of administrative villages (31). Sports federations existed in 72% of counties or higher-level jurisdictions, with a total of 47,280 sports social organizations, including 6,770 youth sports clubs, and over 1.9 million certified social sports instructors (32). However, significant regional disparities and insufficient fiscal guarantees persisted, with many western and rural areas lacking adequate public sport facilities (31).

#### 4.2.3 Micro level: grassroots implementation and cross-sectoral experimentation

Most schools established fitness testing systems and integrated them into evaluation frameworks, though implementation quality varied widely. Studies indicate that primary school students in western counties generally lag behind their urban counterparts in flexibility, speed, and other indicators, and face disadvantages in facility access, teacher availability, and opportunities for organized activities (33). While community sport resources increased, the rate of opening school sports facilities to the public remained only 31.67% (34). Elderly participation in sport showed clear urban-rural disparities (35). Some localities experimented with innovative approaches: in Wuxi, Jiangsu Province, schools opened facilities to the public in designated time slots (36), and in newly urbanized communities, diversified facilities were pre-planned to increase residents' physical activity frequency (37).

Between 1996 and 2015, China's sport and health policy transitioned from a fitness-oriented to a health-oriented framework. At the macro level, strategic objectives were upgraded to align with the national health agenda; at the meso level, institutional frameworks were strengthened and public service coverage expanded; yet at the micro level, participation rates and resource allocation imbalances continued to constrain policy effectiveness.

### 4.3 Collaborative governance and precision intervention (2016–present)

#### 4.3.1 Macro level: strategic upgrading and institutional logic restructuring

The 2016 Healthy China 2030 Planning Outline elevated national fitness and health promotion to a core national development strategy, advancing the principle of “Health in All Policies” and marking a shift from sector-specific sport policy to systemic health governance (38). Coinciding with the consolidation of central authority under Xi Jinping, public health-particularly physical activity-was reframed as integral to national security, economic productivity, and social stability. Subsequent initiatives, including the Healthy China Initiative (2019–2030) and targeted obesity and chronic disease control measures (2020–2024), demonstrated the state's capacity to mobilize multi-sectoral resources in line with top-level priorities. The parallel push for digital governance, aligned with the “Digital China” agenda, reinforced the integration of technological modernization into health governance. This turning point represented not just a policy update but a political recalibration embedding sport-health integration within the Party-state's long-term governance vision.

#### 4.3.2 Meso level: cross-sectoral coordination and digital governance

Government departments in sport, health, education, science and technology, and finance advanced joint policies and resource sharing, institutionalizing the integration of sport and medicine. Multiple regions incorporated exercise prescriptions into community health service systems (18). By the end of 2024, the number of certified social sports instructors had reached ~3.71 million–2.63 per 1,000 people—representing nearly 40% growth since 2015 (39). Digital governance emerged as a critical tool: the National Fitness Program (2021–2025) proposed the creation of a nationwide fitness information platform, enabling interoperability between wearable devices and public health databases (40). Initiatives such as the “Zheliban” fitness module in Zhejiang and “Smart Gym” pilots in Hubei have leveraged data to deliver personalized interventions, while social actors have expanded their roles in event operations, health guidance, and data analytics.

#### 4.3.3 Micro level: enhanced grassroots services and innovative participation models

By the end of 2023, China had 4.5927 million sports venues, with an average of 2.89m^2^ per person (41). Some cities have advanced sport–medicine integration at the community level-for example, Suzhou links community sport centers with medical institutions to deliver exercise prescriptions and chronic disease prevention, while Shanghai has developed a citywide platform to embed physical activity interventions into its health service system (42). Public demand has also shaped policy direction from the bottom up: the “square dance boom” prompted local governments to expand public exercise spaces, and the 2017 Beijing National Fitness Ordinance explicitly called for optimizing urban spatial layouts (43).

Since 2016, China's sport and health policy has achieved a qualitative shift from departmental collaboration to systemic integration. At the macro level, the Healthy China strategy has institutionalized national fitness; at the meso level, cross-sectoral coordination and digital governance have reinforced each other; and at the micro level, public services and participation models have become increasingly targeted and diversified. However, regional disparities and uneven implementation depth remain, providing the empirical basis for the subsequent analysis of institutional tensions.

## 5 Institutional tensions and the evolution of governance logic

The institutional transformation of China's sport and health policy exemplifies the historical institutionalist notion of “change within stability,” whereby gradual adjustments and institutional innovations emerge through the interplay of path dependence, critical junctures, and actor bargaining. As policy goals have expanded from a singular focus on physical fitness to encompass population-wide health, existing institutional structures have revealed adaptive limitations. These constraints have manifested as three core categories of tension-structural, allocative, and behavioral-which both restrict reform capacity and, under certain conditions, serve as drivers of institutional innovation. Together, they have propelled governance logic from a department-led model toward a systemically coordinated approach.

### 5.1 Structural tensions: expanding policy goals and institutional inertia

Despite repeated top-level efforts to promote interdepartmental collaboration under the Healthy China strategy, sustained institutional coordination remains an enduring challenge. This difficulty does not stem from a lack of policy intent. On the contrary, Chinese policymakers have long recognized the importance of cross-sectoral governance and have initiated numerous joint efforts across the sport, health, and education sectors. Nevertheless, the institutional foundations for such collaboration remain relatively underdeveloped.

A core constraint lies in the persistent fragmentation of administrative structures and performance systems. Ministries operate under distinct functional mandates within vertically oriented accountability frameworks that prioritize upward reporting over horizontal coordination. Sectoral performance incentives also diverge significantly: the education system emphasizes exam-based academic outcomes, while the sport and health sectors prioritize physical health indicators and programmatic outputs. These tensions are not unique to China. Even in countries with mature intersectoral platforms, functional silos and limited coordination remain common barriers (5, 6, 44).

In practice, collaborative efforts often take the form of time-limited projects or targeted pilot programs—such as student fitness monitoring or school-based sport-health initiatives—that create short-term momentum but lack routinized, standardized mechanisms for sustained institutionalization (45). Policy resources tend to remain compartmentalized within sectoral boundaries, with limited unified budgeting, integrated performance evaluations, or binding legal mandates for long-term cooperation. Coordination bodies are frequently *ad hoc* in nature, often formed through temporary task forces or joint policy documents without clearly defined responsibilities or stable operating procedures (46). These patterns reflect broader institutional path dependencies that similarly constrain other domains of governance—for example, fragmented financing systems in rural healthcare delivery (47).

As a result, a systemic gap persists between strategic ambition and operational capacity. While top-level plans advocate for life-course health promotion and integrated delivery, institutional compartmentalization and organizational inertia continue to hinder the synergistic implementation of sport and health policies. This recurring governance pattern—marked by “strategic ambition, sectoral fragmentation, and implementation divergence”—does not reflect a failure of vision, but rather the structural difficulty of building sustained, horizontal collaboration within a vertically organized system (48).

### 5.2 Configurational tension: uneven resource distribution and institutional fragmentation

Configurational tension is rooted in the structural imbalances in fiscal investment, infrastructure distribution, and institutional mechanisms for sport and health public services. Although the central government's public service equalization strategy has narrowed urban-rural and regional gaps to some extent-generating positive spatial spillovers in adjacent areas (49)—supply disparities remain significant. As of 2024, China had ~4.8417 million sports venues nationwide, with per capita space around 3.0m^2^ (9, 10). However, regional variation persists: Beijing's per capita venue area was 3.26 m^2^ (50), while Yunnan's stood at around 2.87 m^2^ (51), with notable differences in facility density and maintenance capacity.

These disparities are closely tied to China's fiscal decentralization framework. In vertical fiscal relations, the central government provides transfer payments to underdeveloped regions, but the generalized allocation formulas and lack of performance tracking lead to resource leakage or diversion during local implementation (52). In horizontal fiscal relations, local governments often prioritize projects with high economic returns, viewing public sport and health infrastructure as “soft” expenditures, leading to chronic underinvestment (53). Overall, public sport funding remains low, with a skewed allocation structure-major events and landmark facilities receive priority, while grassroots services and routine maintenance are underfunded, exacerbating regional and urban-rural disparities (54).

Consequently, although the total volume of venues and facilities continues to rise, structural and accessibility gaps persist, failing to meet the diverse needs of different population groups. For example, aging regions lack age-friendly fitness facilities, while newly urbanized districts with high youth populations often lack multifunctional activity spaces. This mismatch between supply and demand structures results in significant differences in the marginal health benefits of public resources across regions, undermining the equity and sustainability of national fitness and health promotion policies.

### 5.3 Behavioral tensions: bargaining and adaptation among diverse actors

Behavioral tensions emerge from the interactions among diverse actors in the implementation of sport and health policy, driven by differences in goal orientation, resource allocation, and jurisdictional boundaries. The Chinese government remains the dominant provider of public sport and health services, steering policy direction through top-level design, fiscal investment, and performance evaluation mechanisms. However, oscillation between “control” and “delegation” has led to cross-sectoral cooperation that is often intermittent and project-based, lacking stable and institutionalized platforms. Encouraging sustained government-market integration can not only preserve strategic direction but also create institutionalized channels for market and societal participation, enhancing the efficiency and sustainability of public service provision (55).

The rise of market forces has introduced technological and model innovations into sport and health services. Fitness chains, sports rehabilitation institutions, and digital platforms (e.g., Keep, Xiaomi Fitness) have demonstrated strong capabilities in data monitoring, exercise prescription, and user engagement (56). However, their business models often clash with the equity goals of public services, high-value services remain less accessible to economically underdeveloped regions and vulnerable populations, potentially exacerbating service disparities.

Social organizations fill gaps in the public system but are often constrained by unstable funding, talent shortages, and limited institutional legitimacy. The public, as both beneficiaries and drivers of health policy, play a dual role: high-quality, accessible public sport spaces can enhance health awareness and community cohesion (57). In recent years, grassroots trends such as the “Village Basketball Association (Village BA)” and “Urban Fun Run” have boosted participation rates and prompted local governments to expand sport spaces, yet have also triggered governance challenges, such as noise complaints and space conflicts.

Overall, behavioral tensions reflect the strategic bargaining among diverse actors within a state-dominated framework. In the absence of long-term cooperative incentives and stable mechanisms for resource integration, actors tend to prioritize departmental or organizational interests, weakening overall policy synergy and trapping cross-sectoral health governance in a low-level equilibrium.

### 5.4 Evolution of governance logic: from mobilization to collaborative precision

**Figure 3:**
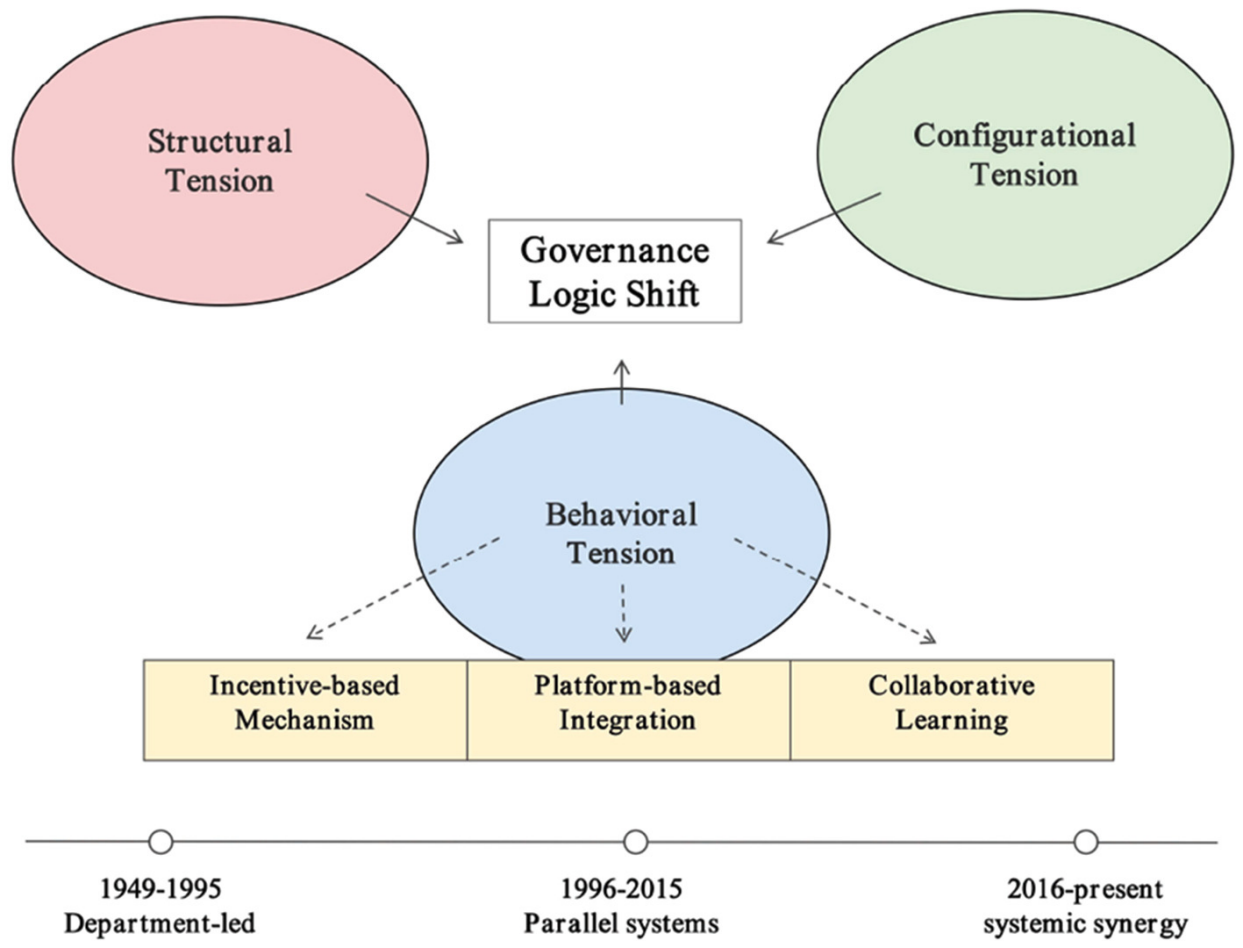
Institutional tensions, adaptive mechanisms, and governance logic shift.

Under the sustained influence of the three types of institutional tension, China's sport and health governance logic has evolved from “single mobilization” to “collaborative precision.” This transformation aligns with the historical institutionalist principle of “change within stability”—whereby old pathways continue to shape policy frameworks while new ideas and tools are embedded at critical junctures, enabling gradual adjustment and structural reconfiguration (see Figure 3).

- From 1949 to 1995, governance logic centered on administrative command and mass mobilization, with the state concentrating resource allocation, narrowly focused goals, and limited actor diversity.
- Between 1996 and 2015, the public health agenda expanded, cross-sectoral platforms and legal frameworks began to emerge, but structural and allocative tensions constrained collaboration, which remained largely confined to short-term projects or targeted campaigns.
- Since 2016, the Healthy China strategy has driven cross-sectoral coordination and digital governance, with the government maintaining strategic leadership while institutionalizing market and social participation. This has fostered a multi-actor governance structure-comprising government, market, society, and the public-characterized by precision interventions and community-embedded services.

This trajectory reflects a shift from department-led governance to a more systemically coordinated model, characterized by recursive interaction across macro, meso, and micro levels. At the macro level, top-level design provides strategic direction and agenda-setting power; at the meso level, institutional platforms integrate cross-sectoral resources and coordinate implementation; and at the micro level, local actors deliver targeted services and foster public engagement based on contextual needs.

Crucially, these levels are not hierarchically separated but mutually constitutive. Micro-level practices not only adapt to existing institutional arrangements but also shape broader governance frameworks through bottom–up experimentation and iterative feedback. Initiatives such as community-based exercise prescriptions and school–community co-management have informed provincial planning and influenced central pilot program design. These dispersed innovations contribute experiential knowledge to higher-level decision-making, consistent with China's policy logic of “experimentation under hierarchy” (58).

In this way, institutional change in China emerges through multi-level feedback loops that connect grassroots experimentation with central recalibration. The evolution of governance thus reflects a dynamic interplay between strategic vision, institutional coordination, and localized adaptation—enabling both policy responsiveness and institutional resilience.

## 6 Conclusion and prospects: institutional reconstruction pathways for China's sport and health policy

### 6.1 Research summary

This study systematically traces the institutional evolution of China's sport and health policy since 1949, revealing a governance logic shift from “enhancing physical fitness” to “health management,” and subsequently to “systemic collaborative governance.” The findings demonstrate that this process has not been one of abrupt replacement, but rather a typical historical institutionalist trajectory: under the constraining inertia of path dependence, policy adjustments and reconstruction have been achieved through breakthroughs at critical junctures combined with incremental, day-to-day modifications.

At the macro level, policy objectives have expanded strategically from a focus on physical fitness to a prioritization of health. At the meso level, governance tools and institutional arrangements have accumulated in layers and undergone periodic recalibration under the influence of institutional tensions. At the micro level, diverse actors have engaged in strategic adaptation and bargaining, reproducing institutions through practice. Overall, China's experience illustrates how a large developing country can achieve a dynamic balance and policy progression between institutional inertia and evolving social demands.

### 6.2 China's international position and comparative value

China's institutional trajectory not only reflects the evolution of its domestic governance logic but also provides a broadly relevant reference for global public health governance-particularly for institutional transitions in developing countries. Facing shared challenges such as the growing burden of non-communicable diseases, fragmented service provision, and structural inequalities, the Chinese model highlights two notable institutional advantages:

- Strategic consistency and cross-sectoral coordination: Through top-level designs such as the Healthy China 2030 and the National Fitness Program, China has ensured policy continuity and integrated institutional execution.
- Technological empowerment and shared responsibility: By leveraging digital platforms, wearable devices, and community pilots, governance has shifted from mass mobilization toward precision intervention.

Compared with South Africa's Community Health Worker program, Brazil's Family Health Strategy, India's ASHA scheme, and nationwide community health network reforms in countries such as Ethiopia and Nepal (59), China has demonstrated stronger state coordination capacity and a higher degree of digital governance integration in advancing universal health coverage. This divergence primarily stems from structural differences in political and administrative contexts: China's centralized political system and unitary administrative structure have enabled top–down institutional integration and rapid digital innovation, whereas South Africa's federal health system and the political instability in some countries have, to some extent, weakened policy coherence and inter-agency coordination capacity. Such a comparison not only underscores the critical role of political capacity and institutional cohesion in shaping viable governance pathways but also suggests that, in promoting the Chinese experience to other developing countries, careful assessment of the compatibility and transferability of political and administrative systems is essential.

### 6.3 Policy recommendations

Drawing on the analysis of institutional tensions, future optimization and reconstruction of China's sport and health policy could proceed in three directions:

- Enhancing institutional integration capacity: Establish permanent cross-departmental coordination mechanisms, promote data and information sharing across sport, health, and education sectors, and build a unified health governance framework.
- Strengthening grassroots governance units: Develop “micro-governance platforms” such as school-based health stations and community sport centers to improve accessibility, adaptability, and professionalism in policy implementation, ensuring targeted local delivery.
- Deepening digitalization and data-driven governance: Integrate wearable devices, big data, and artificial intelligence to build health archives and early-warning systems covering the entire life course, facilitating a transition from universal interventions to precision governance.

### 6.4 Research limitations and future directions

This study is based on 15 national-level policy documents and provides a stage-based analysis of macro-level institutional logic. However, it does not fully capture local policy dynamics and regional disparities, potentially underestimating the role of bottom-up institutional innovation. While textual analysis has revealed the semantic dimensions of policy and institutional change, it remains limited in empirically examining actor networks and interest structures. Future research could extend in two directions:

- Combining local case studies, cross-national comparisons, and social network analysis to present a more comprehensive picture of the multi-level governance ecosystem.
- Advancing theoretical innovation by integrating historical institutionalism with complex systems theory and adaptive governance models, thereby deepening understanding of evolutionary mechanisms under uncertainty and institutional resilience.

In sum, the institutional evolution of China's sport and health policy offers both an empirical example of how developing countries address structural and allocative tensions, and an institutional framework for global health governance transformation. As “health promotion” becomes an increasingly shared global consensus, an in-depth institutional analysis of the Chinese experience not only contributes to fostering North-South knowledge dialogue and mutual learning but also provides a distinctive “China solution” for building an inclusive, multi-level, and sustainable global health governance system.

## Data Availability

The raw data supporting the conclusions of this article will be made available by the authors, without undue reservation.
